# Honey Bee Inhibitory Signaling Is Tuned to Threat Severity and Can Act as a Colony Alarm Signal

**DOI:** 10.1371/journal.pbio.1002423

**Published:** 2016-03-25

**Authors:** Ken Tan, Shihao Dong, Xinyu Li, Xiwen Liu, Chao Wang, Jianjun Li, James C. Nieh

**Affiliations:** 1 Key Laboratory of Tropical Forest Ecology, Xishuangbanna Tropical Botanical Garden, Chinese Academy of Science, Kunming, Yunnan Province, China; 2 Eastern Bee Research Institute, Yunnan Agricultural University, Heilongtan, Kunming, Yunnan Province, China; 3 Section of Ecology, Behavior, and Evolution, Division of Biological Sciences, University of California, San Diego, La Jolla, California, United States of America; Queen Mary University of London, UNITED KINGDOM

## Abstract

Alarm communication is a key adaptation that helps social groups resist predation and rally defenses. In Asia, the world’s largest hornet, *Vespa mandarinia*, and the smaller hornet, *Vespa velutina*, prey upon foragers and nests of the Asian honey bee, *Apis cerana*. We attacked foragers and colony nest entrances with these predators and provide the first evidence, in social insects, of an alarm signal that encodes graded danger and attack context. We show that, like *Apis mellifera*, *A*. *cerana* possesses a vibrational “stop signal,” which can be triggered by predator attacks upon foragers and inhibits waggle dancing. Large hornet attacks were more dangerous and resulted in higher bee mortality. Per attack at the colony level, large hornets elicited more stop signals than small hornets. Unexpectedly, stop signals elicited by large hornets (SS _large hornet_) had a significantly higher vibrational fundamental frequency than those elicited by small hornets (SS _small hornet_) and were more effective at inhibiting waggle dancing. Stop signals resulting from attacks upon the nest entrance (SS _nest_) were produced by foragers and guards and were significantly longer in pulse duration than stop signals elicited by attacks upon foragers (SS _forager_). Unlike SS _forager_, SS _nest_ were targeted at dancing and non-dancing foragers and had the common effect, tuned to hornet threat level, of inhibiting bee departures from the safe interior of the nest. Meanwhile, nest defenders were triggered by the bee alarm pheromone and live hornet presence to heat-ball the hornet. In *A*. *cerana*, sophisticated recruitment communication that encodes food location, the waggle dance, is therefore matched with an inhibitory/alarm signal that encodes information about the context of danger and its threat level.

## Abstract 2

Honey bees possess a vibrational alarm signal that encodes information about the level of danger at food and at the nest entrance.

## Author Summary

Asian honey bees are attacked by formidable predators, giant hornets, at food sources and at their nests. We show that one species of honey bee, *Apis cerana*, has evolved an alarm signal, the stop signal, which warns nestmates of this danger. The stop signal consists of a brief vibrational pulse that encodes information about the danger level in signal frequency and the danger context in signal duration. Each danger level and danger context results in a distinctive signal. Receivers of these signals respond appropriately, stopping recruitment for the dangerous food location or remaining inside the safety of the nest, according to the encoded danger level and context. This is the first known example of such a complex alarm signal in an insect and demonstrates a new level of sophistication in bee communication.

## Introduction

Animals have evolved sophisticated abilities to communicate predator threats. These warning signals are found in a wide range of organisms, such as insects, birds, and primates [1–3]. In some vertebrates, these signals can be referential, encoding information about objects or events external to the signaler, such as the identity of the predator, and eliciting appropriate responses [4–6]. More commonly, individuals can increase signaling or modify their signals to indicate threat urgency. Chuck alarm calls in Richardson’s ground squirrel (*Spermophilus richardsonii*) increase when the alarm stimulus is closer and elicit greater vigilance [7]. Modulation of individual signals or varying call sequences also occurs. Predators that are closer, larger, and faster elicit chicken alarm calls that are shorter, louder, and lower in frequency [2]. Mexican chickadees (*Parus sclateri*) give higher-pitched alarm calls in riskier situations [8]. Redfronted lemur (*Eulemur fulvus rufus*) alarm vocalizations increase in frequency in response to higher threat arousal levels, and higher frequencies elicit longer orienting responses [3]. Squirrel monkeys (*Saimiri sciureus*) also increase call frequencies in response to greater threats, and higher frequency or louder calls also result in longer orientation responses [9]. Alarm calls of the social mongoose (*Suricata suricatta*) vary acoustically depending upon the predator type and urgency level [10]. However, to date, no such graded individual alarm signals have been identified in social insects, although many social insect species are highly cooperative and depend upon signaling to coordinate key aspects of their collective behavior [11–17].

Nearly all social insects can use alarm pheromones to communicate danger [1]. Termites [15], treehoppers [16], and ants [17] can also use vibrations to signal alarm. However, there is no evidence that these individual signals are graded according to threat level, perhaps because the collective response of the colony provides the overall, finely tuned response, with multiple alarm signals additively reflecting the level of danger and eliciting an appropriate reaction.

The European honey bee, *Apis mellifera*, does possess an inhibitory signal, the stop signal, which can be elicited by peril to reduce recruitment to a dangerous location [18]. For example, *A*. *mellifera* foragers produce stop signals to inhibit waggle dancing [19–21] for dangerous food sources. A stop signal is a 300–400 Hz vibrational signal with a duration of approximately 150 ms [20,22] that a worker usually delivers while butting its head into the body of the receiver, causing the receiver to momentarily freeze [12,19,23,24]. Attacks by wasp and spider predators [12] and conspecifics [18] elicit stop signals, which inhibit waggle dancing. However, stop signals are also used in other inhibitory contexts that are not associated with danger. House-hunting bees speed up nest site decision-making with stop signals that inhibit recruitment for competing nest sites [20]. Food source overcrowding also triggers stop signaling [19,22,25]. In *A*. *mellifera*, the stop signal is thus best understood as an inhibitory signal that reduces waggle dancing. Nevertheless, stop signals may possess additional functions if they occur in other bee species, particularly if there is an evolutionary need for a warning signal against common and deadly predators.

**Fig 1:**
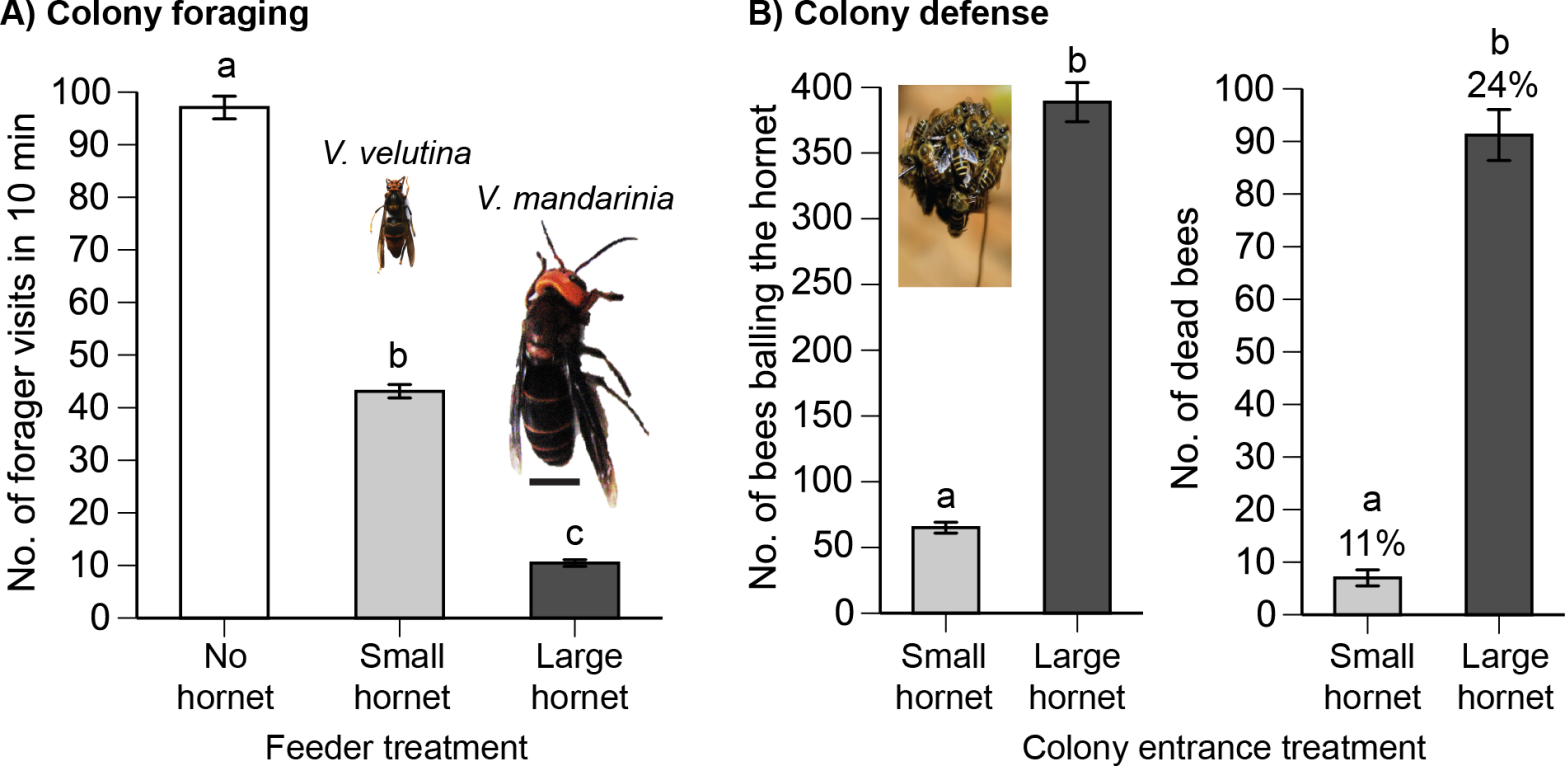
Effects of hornet attacks upon colony foraging allocation and defense. In both contexts, larger hornets were more dangerous. (A) The large hornet elicited the greatest colony foraging aversion (six trials/treatment/colony for a total of 54 trials). Scale bar is 1 cm. Different letters indicate significant differences (Tukey’s Honestly Significant Difference [HSD] test, *p* < 0.05). (B) When it attacked the colony, the large hornet was balled by 6-fold more bees, and 13-fold more of these bees died (percentage dead also shown, three trials/treatment/colony for a total of 18 trials). An image of bees balling the small hornet is shown. Different letters indicate significant differences. Graphs show the mean ± 1 standard error.

The Asian honey bee species, *Apis cerana*, is an excellent model for studying the effects of predator threats on colony signaling, because these bees require a coordinated defense against common hornet predators. *A*. *cerana* occurs throughout southern and eastern Asia, with a geographic range extending from India to China and Japan [26]. Throughout this range, hornets such as *Vespa velutina* [27] and the larger hornet, *Vespa mandarinia* (Fig 1), can attack *A*. *cerana* foragers (Tan, personal observation) and directly attack the nest [28–30]. These nest attacks pose a severe danger to colony survival, because the armor of both hornet species resists bee stings. Hornets typically wait outside the nest entrance, killing nest defenders until they are depleted, and then enter the nest to harvest the brood [29]. Up to 30% of a colony’s workers can die during a single attack upon a poorly defended colony [31]. *Apis cerana* has, therefore, evolved a remarkable strategy of forming a ball of bees around the attacker and heating it to death [29]. It is unclear exactly what triggers heat-balling, though alarm pheromone [31] and, potentially, other signals may be involved. We thus chose to study *A*. *cerana*, a native honey bee species that is also an important pollinator of agricultural and native Asian plants [32,33].

Hornets attack *A*. *cerana* in two contexts: during foraging and at the nest. We therefore tested if workers would produce stop signals in both situations. We hypothesized that predators posing a greater threat to individuals or the colony would elicit stop signals with graded changes in duration, frequency, or both. Our goals were to determine (1) if *A*. *cerana* has a “stop signal” that inhibits waggle dancing, (2) if such stop signals are elicited by attacks upon foraging bees and the nest, (3) if a more dangerous predator changes signaling characteristics and signal number, (4) if these signals are referential, and (5) if stop signals influence heat-balling.

## Results

We tested the behavior of three *A*. *cerana* observation colonies by using tethered hornets to attack (1) free-flying foragers trained to a rich sucrose solution feeder or (2) bees at the nest entrance. We tested only one colony with one treatment at a time: large hornet, small hornet, or control. We video recorded bee behaviors inside the nest and recorded bee sounds with a directional electret microphone. In each experiment, treatments influenced all three colonies in similar ways. We therefore show the pooled responses of the colonies and report the colony effect size.

### The Larger Hornet Was a Greater Threat

The large hornet (*V*. *mandarinia*) elicited stronger forager aversion (Fig 1A) and greater colony mortality (Fig 1B) than the small hornet (*V*. *velutina*). Colonies allocated foraging according to predator danger (*F*_2,49_ = 834.26, *p* < 0.0001, all pairwise differences significantly different, Tukey’s Honestly Significant Difference [HSD] test, *p* < 0.05).

During nest attacks, 6-fold more bees heat-balled the large hornet as compared to the small hornet (*F*_1,14_ = 399.45, *p* < 0.0001), which is what one would expect given that the surface area of an ellipsoid surrounding a hornet is 6-fold greater for the large (0.91 cm^2^) as compared to the small hornet (0.15 cm^2^). The larger hornet resulted in greater bee mortality: 13-fold more bees died in the heat ball around the large hornet as compared to the small hornet (*F*_1,16_ = 275.83, *p* < 0.0001).

### Foragers That Were Attacked Reduced Waggle Dancing and Produced Stop Signals

**Fig 2:**
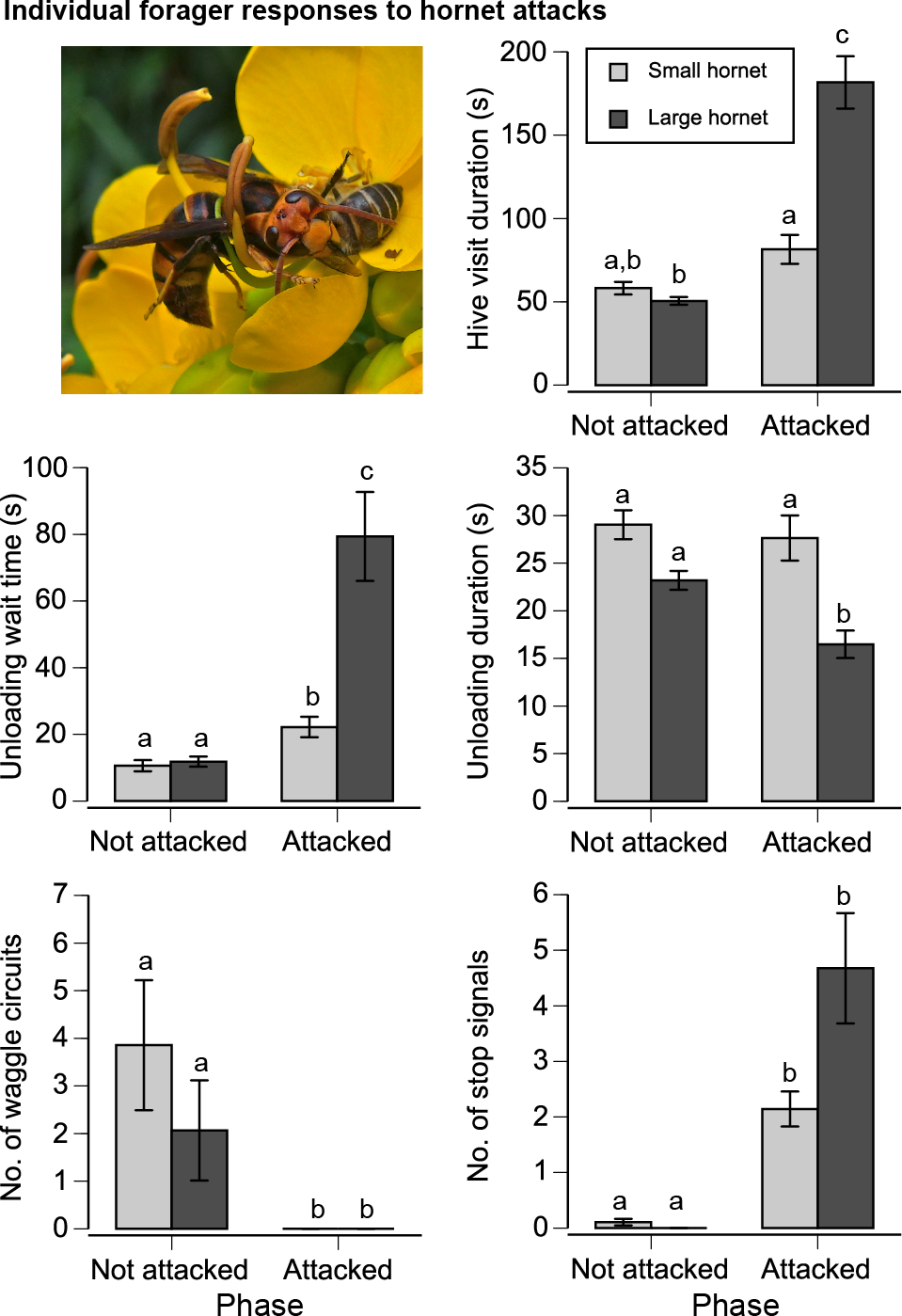
Effect of small and large hornet attacks at the food source on the individual behavior of foragers in the nest (from three bee colonies, 56 bees attacked by small hornet and 72 bees attacked by large hornet). Standard error bars are shown. Different letters indicate significant differences (Tukey’s HSD test, *p* < 0.05). Photo shows *V*. *velutina* (small hornet) killing an *A*. *cerana* forager on a *Cassia surattensis* inflorescence.

Foragers that were attacked by the large but not the small hornet remained in the nest significantly longer upon their return (attack phase effect: *F*_1,57_ = 93.58, *p* < 0.0001) as compared to when they were not attacked (Fig 2). Treatment (hornet type effect: *F*_1,55_ = 20.25, *p* < 0.0001) and the interaction treatment*phase (*F*_1,57_ = 36.57, *p* < 0.0001) were significant, and the colony accounted for 3% of model variance.

Similarly, foragers attacked by the large hornet waited longer before unloading their food (phase effect: *F*_1,57_ = 76.04, *p* < 0.0001, 10% colony effect). Treatment was significant (*F*_1,55_ = 22.41, *p* < 0.0001), and the interaction treatment*phase was significant (*F*_1,57_ = 9.87, *p* = 0.003). Foragers attacked by the large hornet also spent less time unloading their collected food (phase effect: *F*_1,57_ = 14.35, *p* = 0.0004). Treatment (*F*_1,55_ = 30.42, *p* < 0.0001) and the interaction treatment*phase (*F*_1,57_ = 8.42, *p* = 0.005) were significant (9% colony effect, Fig 2).

Attacks reduced waggle dancing (phase effect: *F*_1,57_ = 18.97, *p* < 0.0001. Treatment and the interaction treatment*phase were not significant (<1% colony effect, Fig 2).

Attacks by both hornet species elicited stop signals. A stop signaler typically produced a vibrational signal by lunging at a receiver and making contact with its head against the receiver. All receivers were motionless while the vibrations were delivered, exhibiting the classic freezing response seen in *A*. *mellifer*a [12]. There was a significant effect of phase (*F*_1,57_ = 96.55, *p* < 0.0001), but treatment and the interaction treatment*phase were not significant (12% colony effect, Fig 2). Although large hornets appeared to elicit more stop signals per forager than small hornets, this difference was not significant (Tukey’s HSD test, *p* > 0.05).

### Colony Stop Signaling Increased According to Predator Size

**Fig 3:**
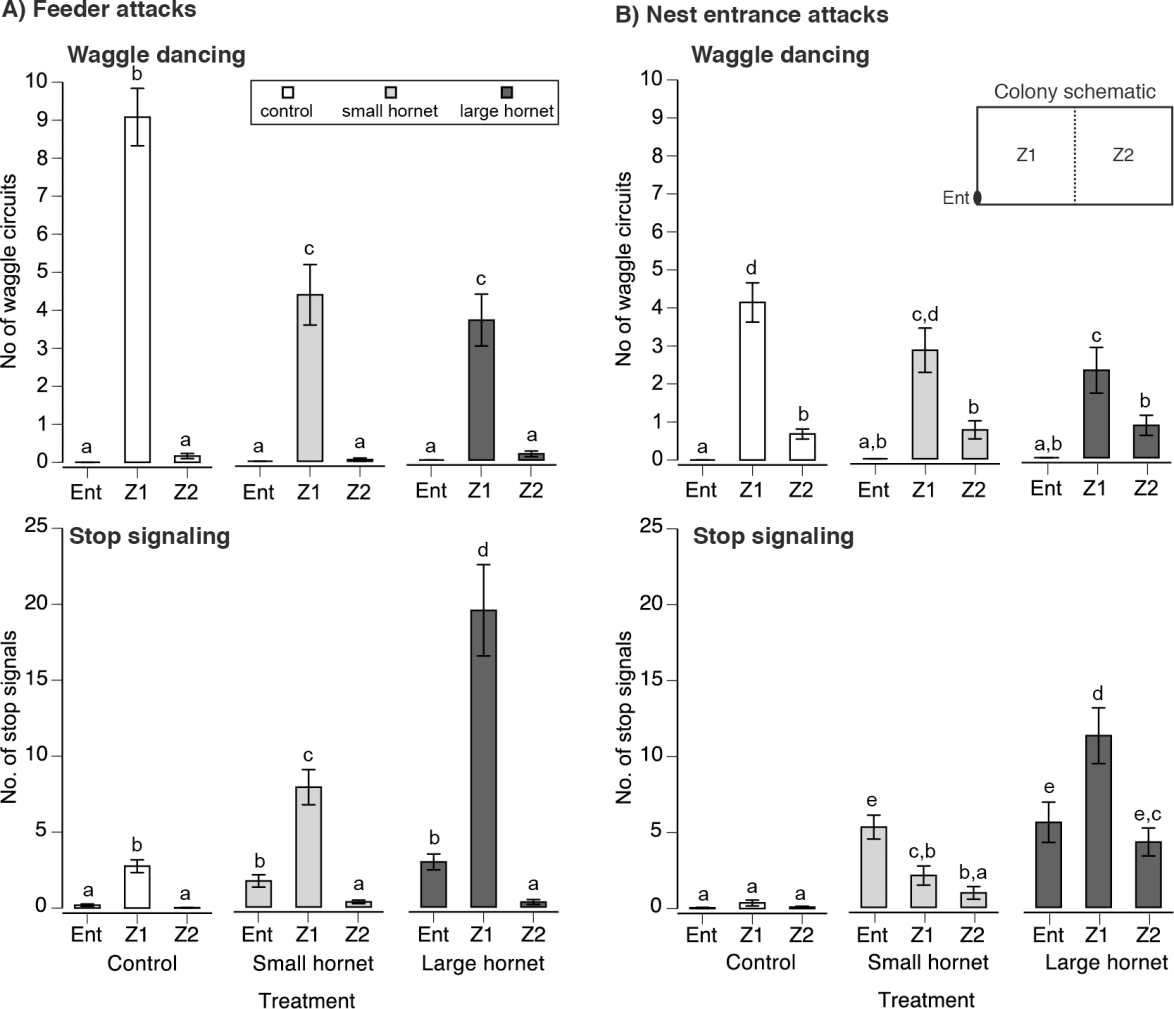
The effect of hornet attacks upon (A) foragers (49 trials: 17, 16, and 16 from colonies 1, 2, and 3, respectively) and (B) at the nest entrance upon stop signaling and waggle dancing (60 trials: 20, 22, and 18 from colonies 1, 2, and 3, respectively). The colony schematic shows the location of the nest entrance (Ent) and the two comb zones (Z1 = dance floor) and (Z2). Graphs show the mean ± 1 standard error. Different letters indicate significant differences (Tukey’s HSD test, *p* < 0.05).

We then examined the effects of predator attack on colony-level signaling. When hornets attacked foragers at the feeder (Fig 3A), colony level waggle dancing decreased by 0.5-fold (treatment: *F*_2,93_ = 12.55, *p* < 0.0001). There was a significant effect of location (feeder versus nest attacks: *F*_2,190_ = 347.68, *p* < 0.0001) and the interaction treatment*location (*F*_4,190_ = 11.06, *p* < 0.0001, 9.3% colony effect) because most dancing occurred on the dance floor (Z1). Large and small hornet attacks equally reduced waggle dancing (Tukey’s HSD test, *p* > 0.05).

Attacks upon foragers increased colony-level stop signal production (treatment: *F*_2,93_ = 83.73, *p* < 0.0001). Stop signaling occurred primarily on the dance floor (Z1), and, thus, there was a significant effect of location (*F*_2,190_ = 226.38, *p* < 0.0001) and the interaction treatment*location (*F*_4,190_ = 17.67, *p* < 0.0001, <1% colony effect). Attacks by the large hornet upon foragers elicited 7.7-fold more stop signals (SS _large hornet forager_) than the control treatment and 2.3-fold more stop signals than those elicited by the small hornet (SS _small hornet forager_, Tukey’s HSD test, *p* < 0.05, Fig 3A).

Colony attacks by hornets elicited similar changes (Fig 3B). Most waggle dancing occurred on the dance floor (significant location effect: *F*_2,158_ = 102.49, *p* < 0.0001). Nest attacks by the large hornet decreased colony waggle dancing by 0.4-fold as compared to the control treatment (Tukey’s HSD test, *p* < 0.05), but the control and small hornet treatments were not significantly different (Tukey’s HSD test, not significant [NS]). There was no significant effect of treatment, but the nearly significant interaction treatment*location (*F*_4,158_ = 2.25, *p* = 0.07, 0.5% colony effect) reflected the Tukey’s HSD test results.

Interestingly, colony attacks also elicited stop signaling (treatment: *F*_2,77._ = 156.63, *p* < 0.0001, Fig 3B). Attacks by both hornets upon the nest increased stop signaling by 111-fold as compared to the control no-attack phase. Overall, attacks by the large hornet elicited 2.4-fold more stop signals than the small hornet, and 20.2-fold more signals than the control treatment (Tukey’s HSD test, *p* < 0.05). There was a significant effect of location (*F*_2,158_ = 19.98, *p* < 0.0001) and the interaction treatment*location (*F*_4,158_ = 15.22, *p* < 0.0001, <1% colony effect). Stop signals elicited by the small hornet attacking the nest (SS _small hornet nest_) occurred mainly in the nest. Stop signals elicited by the large hornet attacking the nest (SS _large hornet nest_) covered a wider range and occurred on the dance floor and in the nest.

### Stop Signals Elicited by Different Predators Differentially Inhibited Waggle Dancing

**Fig 4:**
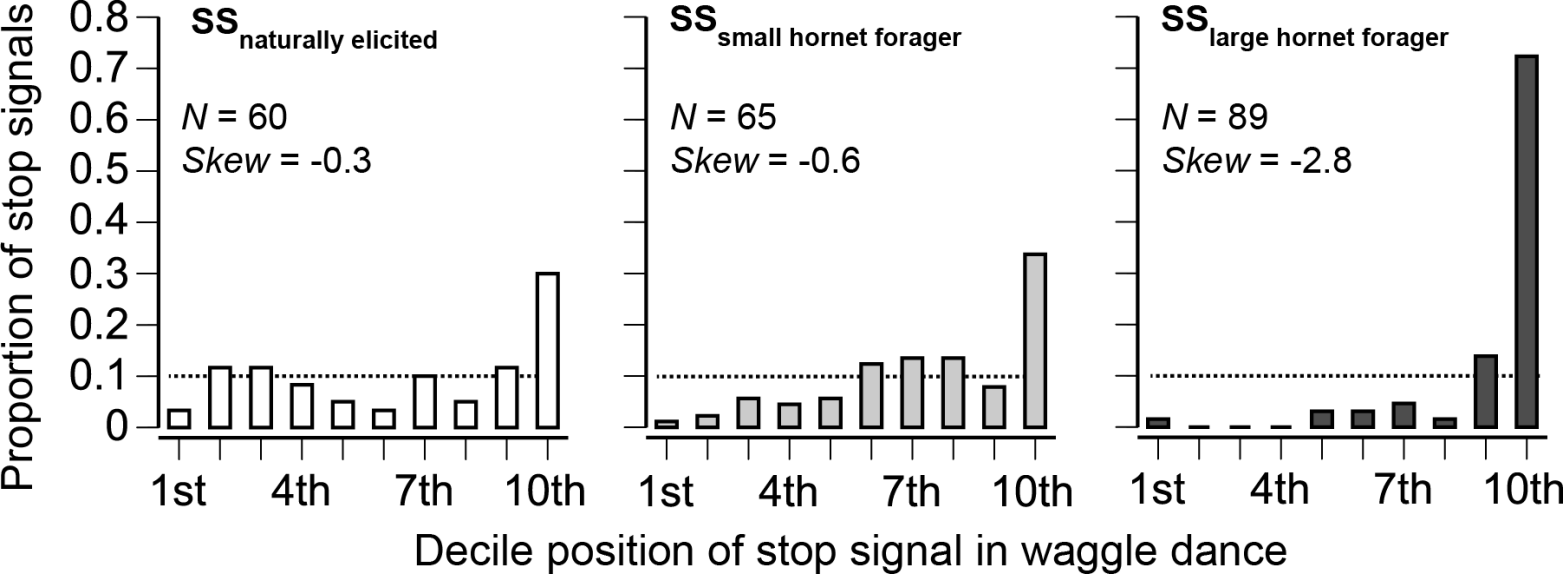
Effect of stop signals (SS) elicited by unknown natural causes, the small hornet, and the large hornet upon waggle dancing (dances performed by 51, 58, and 51 different bees from three colonies; the number of stop signals is shown in each plot). The effect of naturally elicited stop signals was observed with bees waggle dancing for natural food sources. All other dances were from bees trained to a feeder, none of which directly experienced the predator. If stop signals had no effect upon waggle dancing, they would be uniformly distributed (shown by a dashed line). However, all distributions are significantly different from a uniform distribution (*p* << 0.00001). As the efficacy of the inhibitory signal increases, the distributions should become more negatively skewed. A stop signal that always immediately stops waggle dancing would have only a single bar at the tenth decile. Stop signals produced by foragers attacked by the large hornet (SS _large hornet forager_) were significantly more inhibitory (*p* << 0.00001) than those elicited by the small hornet (SS _small hornet forager_).

The stop signals produced by *A*. *cerana* inhibited waggle dancing (Fig 4). We first examined bees waggle dancing for natural food sources that received stop signals elicited by unknown natural causes (SS _naturally elicited_): 42% of these waggle dances ended with a stop signal in the last two deciles of the dance (significantly different from a uniform distribution, *χ*_*9*_^2^ = 33, *p* = 0.0001).

We then conducted a bee-produced stop signal playback experiment. We trained bees to sucrose feeders and tested the effect of stop signals on waggle dancers who were not exposed to the predator. These waggle dancers were not attacked: they were recruiting for a nearby, same-scented feeder and, therefore received stop signals [18]. Such waggle dancers also stopped dancing after receiving stop signals elicited by small hornet (*χ*_*9*_^2^ = 71.56, *p* << 0.0001^SB^) or large hornet (*χ*_*9*_^2^ = 276.40, *p* << 0.0001^SB^) attacks. Crucially, stop signals elicited by the large hornet (SS _large hornet forager_) were significantly more inhibitory than those elicited by the small hornet (SS _small hornet forager_, *χ*_*9*_^2^ = 90.36, *p* << 0.0001^SB^). The skew, a measure of stop signal effectiveness, was 5-fold greater in magnitude for SS _large hornet forager_ as compared to SS _small hornet forager_ (Fig 4).

### Predator and Attack Context Altered Stop Signal Vibrational Characteristics

**Fig 5:**
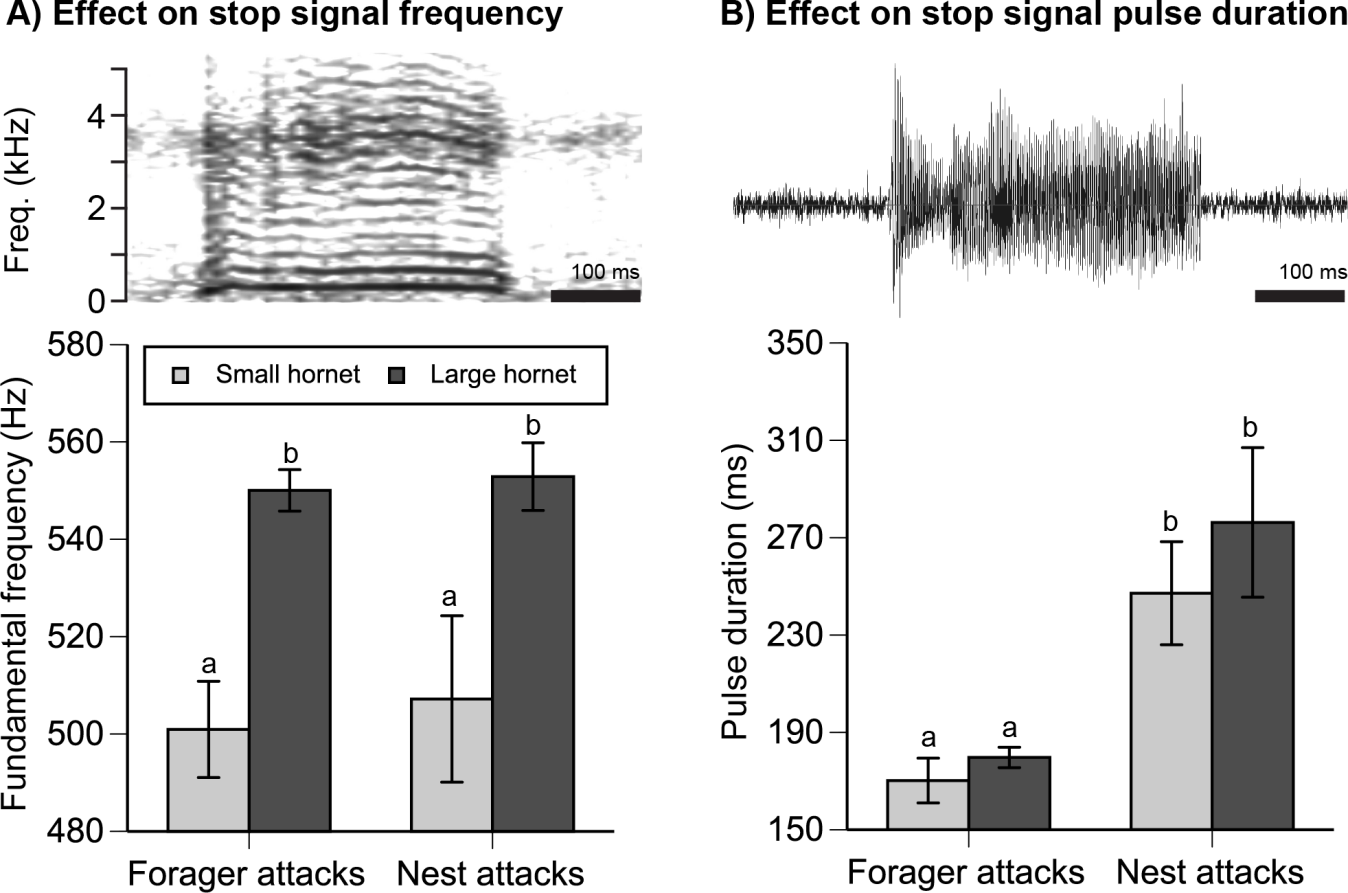
Effect of predator type upon stop signal vibrational characteristics (*n* = 282 bees from three colonies, analysis based upon the mean stop signal frequency and pulse duration per bee). (A) Large hornets elicited stop signals with a consistently higher fundamental frequency. The spectrogram shows a typical stop signal produced by a forager attacked by the large hornet. (B) Nest attacks elicited signals with a consistently longer pulse duration. The oscillogram shows a typical stop signal produced by a worker attacked by the hornet at the nest entrance. Graphs show the mean ± 1 standard error. Different letters indicate significant differences (Tukey’s HSD test, *p* < 0.05).

The stronger inhibitory effect of SS _large hornet forager_ as compared to SS _small hornet forager_ corresponded to significant differences in signal fundamental frequency (Fig 5A). The vibrational fundamental frequency of stop signals was 47 Hz higher for the large as compared to the small hornet (attacker effect: *F*_2,279_ = 17.83, *p* < 0.0001; experiment and attacker*experiment interaction NS, 21% colony effect). Hornet attacks upon the nest elicited 1.4-fold longer stop signals than hornet attacks on bees at a feeder (experiment effect: *F*_1,279_ = 35.28, *p* < 0.0001, Fig 5B). However, stop signal durations were not significantly influenced by predator type (attacker effect: *F*_1,279_ = 3.39, *p* = 0.07, attacker*experiment interaction NS, 12% colony effect).

### Guard Bees and Foragers Produced Stop Signals during Nest Attacks and Receivers Stayed Inside the Nest

**Fig 6:**
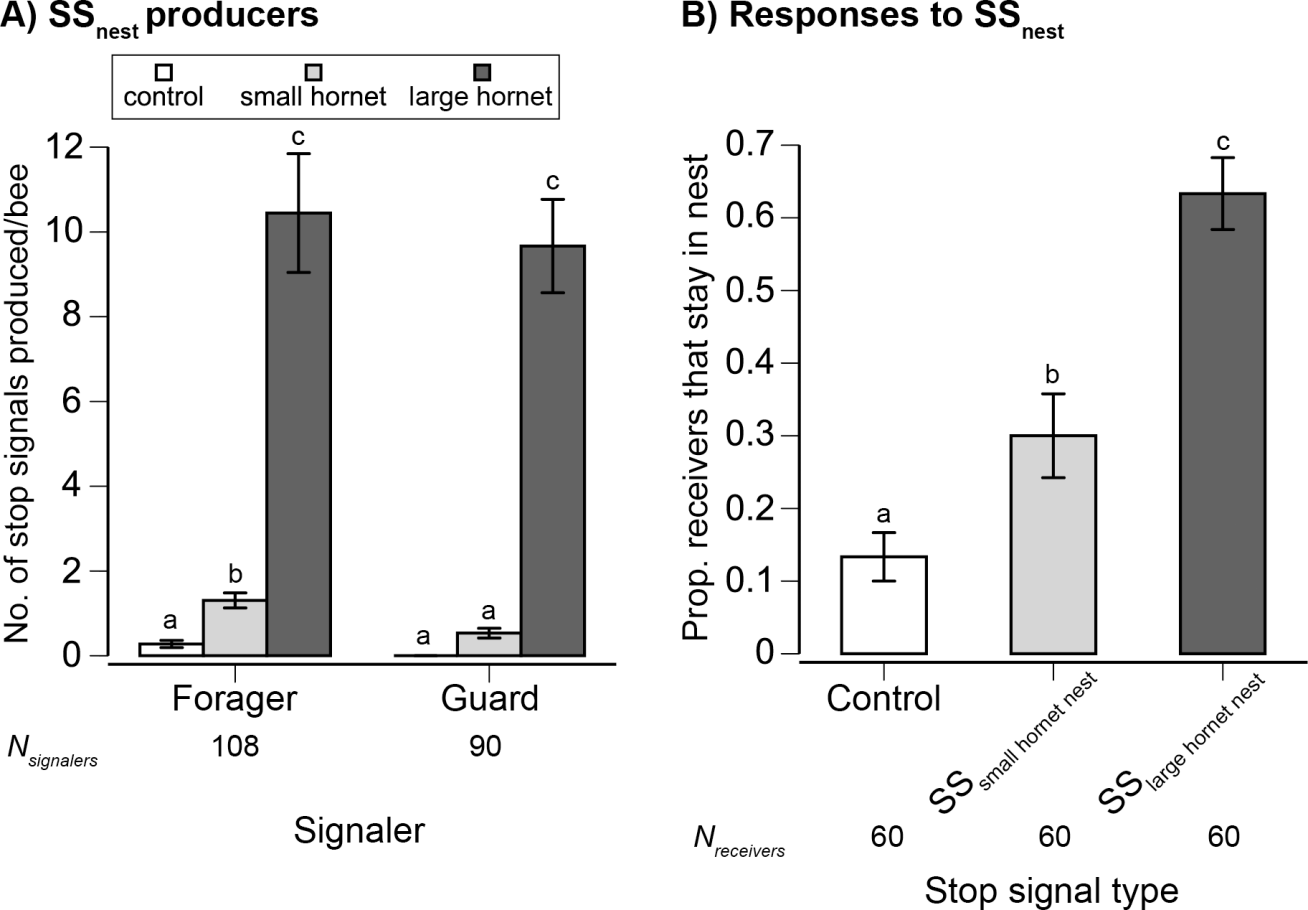
The effect of nest attacks upon (A) stop signal (SS _nest_) production by foragers and guards. (B) Receivers of SS _nest_ increasingly remained inside the nest according to the type of predator eliciting the signal. Graphs show the mean ± 1 standard error. Different letters indicate significant differences (Tukey’s HSD test, *p* < 0.05).

Colony entrance attacks triggered stop signaling by returning foragers and guard bees (treatment effect: *F*_2,190_ = 344.19, *p* < 0.0001, Fig 6A). We marked these bees with colored starch powder so that we could track them inside the nest. None of these bees came into physical contact with the hornet, but could see and smell its presence at the nest entrance. The large hornet elicited 8-fold to 18-fold more stop signals from foragers and guards, respectively, than the small hornet (Tukey’s HSD test, *p* < 0.05). The small hornet elicited 2.4-fold more stop signals per bee from foragers than from guards, leading to a significant effect of worker type (*F*_1,190_ = 6.98, *p* = 0.009). The interaction treatment*worker type was not significant (*F*_2,190_ = 2.73, *p* = 0.07, <1% colony effect).

Receivers of these stop signals did not go out to attack the hornet. Instead, they increasingly stayed inside the nest according to the threat level communicated by stop signals (Fig 6B). To avoid disturbing stop signal receivers, we used different colors of starch powder to pre-mark a number of foragers (bees carrying pollen or nectar) as they entered the nest and before we presented the treatment (control, small hornet, or large hornet) at the nest entrance. All stop signal receivers were inside the nest when the treatment was presented and, therefore, had no direct knowledge of the treatment. Interestingly, all stop signals elicited by nest attack were received by foragers, some of which were waggle dancing inside the nest. Signalers did not target guard bees. SS _large hornet nest_ and SS _small hornet nest_ respectively increased the probability of foragers remaining inside the nest by 4.8-fold and 2.3-fold as compared to control bees (treatment effect: *F*_2,13_ = 33.21, *p* < 0.0001, 18% colony effect). All treatments resulted in significantly different responses (Tukey’s HSD test, *p* < 0.05).

### Heat-balling Was Elicited by Sting Alarm Pheromone and Predator Presence, Not Stop Signals

**Fig 7:**
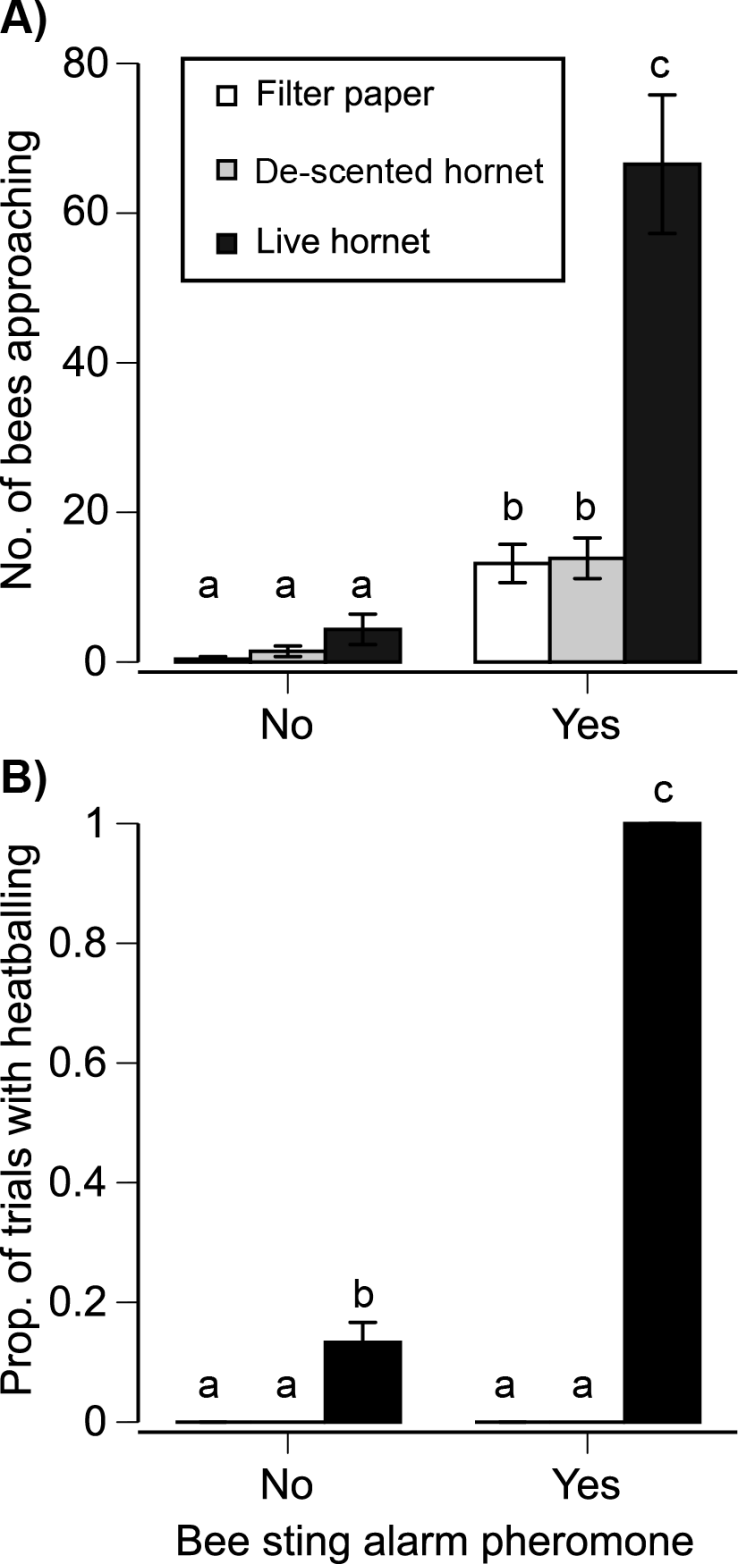
Stimuli that elicit (A) approaches to and (B) heat-balling of the small hornet (*V*. *velutina*). The alarm pheromone (one bee sting equivalent) was presented at the beginning of each trial (ten trials/colony for 30 total trials). Graphs show the mean ± 1 standard error: 100% of live hornets presented with honey bee sting alarm pheromone were heat-balled (0 standard error for this treatment). Different letters indicate significant differences (Tukey’s HSD test, *p* < 0.05).

What triggered heat-balling? We compared colony responses to three types of targets presented at the nest entrance: clean filter paper, a dead small hornet cleaned of cuticular hydrocarbon odors, and a live small hornet. Bees could add sting alarm pheromone during the attack process, but we also tested the effect of initially presenting alarm pheromone (resulting in six treatment types). There was a significant effect of treatment on bee approaches to the hornet (*F*_5,172_ = 361.88, *p* < 0.0001) such that the live hornet with alarm pheromone elicited a 5-fold to 166-fold greater response than any other treatment (Tukey’s HSD test, *p* < 0.05, Fig 7A). There was no significant colony effect on approaches (*F*_2,172_ = 0.92, *p* = 0.40). Similarly, there was a significant effect of treatment on bee heat-balling of the hornet (*F*_5,10_ = 868.00, *p* < 0.0001, Fig 7B). Bees would heat-ball a live hornet (but no other targets) regardless of alarm pheromone presence. However, alarm pheromone strongly increased heat-balling of live hornets by 7.7-fold. There was no significant effect of colony identity on heat-balling (*F*_2,10_ = 1, *p* = 0.40). In all cases, the dead hornet cleaned of odors elicited the same responses as the clean filter paper (Fig 7, Tukey’s HSD tests, *p* < 0.05). Thus, hornet odor and honey bee sting alarm pheromone were the main attack triggers, with alarm pheromone eliciting the strongest effect.

## Discussion

We provide the first evidence, in a social insect, of a functionally referential alarm signal in which individual signals are altered according to danger context (pulse duration) and predator type (fundamental frequency). Attacks by important natural predators, hornets, on *A*. *cerana* foragers significantly increased the production of a foraging-context stop signal that inhibited waggle dancing according to predator danger level. Attacks by hornets upon the nest entrance elicited distinctive stop signals that inhibited forager departure from the safe nest. We call these vibrational signals “stop signals” because their general physical characteristics, fundamental frequency and duration, are similar to the stop signal of *A*. *mellifera* and because they elicited the same immediate response: receivers froze during signal delivery.

The large hornet (*V*. *mandarinia*) posed a higher threat because it elicited more foraging aversion and nest defender mortality than the small hornet (*V*. *velutina*, Fig 1). Because of high variation in individual signal production, there was no significant difference in the number of stop signals elicited per forager by the different predators attacking bees at the feeder, although the large predator elicited 2.2-fold more signals (Fig 2). Colonies similarly produced 2.3-fold to 2.5-fold more stop signals in response to attacks by the large as compared to the small predator upon foragers and at the nest. However, this difference was significant (Fig 3) due to reduced variation in colony responses.

Our data suggest that stop signals are only triggered by personal information about danger. If receiving a stop signal (but having no direct experience of peril) was sufficient to elicit signaling, there should have been a chain reaction that increased the number of stop signals. However, this did not occur. Foragers attacked by small and large hornets respectively produced an average of two and five stop signals per bee (Fig 2). To measure the colony response, we attacked five foragers at a time with the small or large hornet and respectively measured an average of ten and 23 stop signals in the colony (Fig 3A), almost precisely what one would expect if only the attacked bees signaled.

Large and small hornet attacks upon foragers (Fig 3) significantly decreased colony waggle dancing by the same degree. In the feeder experiment, this likely occurred because most waggle dancers were recruiting for the feeder and directly reduced their dancing when attacked. Thus, the inhibitory signal was tuned to the threat level, but decreases in the excitatory signal were not. This result differs from McCollough and Nieh [12], who used wasp and spider predators that elicited similar overall responses in *A*. *mellifera* foragers, but found no tuning according to attack severity or bee response severity. In contrast, we studied *A*. *cerana* and used predators that elicited significantly different levels of foraging aversion, likely because of their much greater natural size differences (Fig 1A). Species and predator threat differences may explain why *A*. *cerana* tuned stop signal production to predator threat level.

Only nest attacks by the large hornet reduced waggle dancing compared to the control treatment (Fig 3B). This decline in waggle dancing is expected because stop signals elicited by nest attacks were all targeted at foragers, and SS _large hornet_ more strongly inhibited waggle dancing than SS _small hornet_ (Fig 4). Nest attacks by the large hornet also more strongly elevated stop signaling on the dance floor than nest attacks by the small hornet (Fig 3B).

We hypothesized that SS _nest_ would cause workers to attack the hornet. Instead, all SS _nest_ were targeted at foragers and increased their probability of remaining inside the nest, scaled to the peril outside (Fig 6B). Thus, the targets of SS _nest_ and SS _forager_ are evidently foragers. Whereas SS _forager_ were targeted at waggle dances and stopped waggle dancing, SS _nest_ were received by both dancing and non-dancing foragers and resulted in a common response: inhibiting departure from the safer interior of the nest.

Interestingly, both foragers and guards can produce SS _nest_. This provides the first evidence that stop signals may be produced by other worker types, not just foragers [18,34] or nest-site scouts [20]. Many SS _nest_ were delivered inside the nest entrance tube (Fig 3B), which was too narrow for waggle dancing. Such stop signaling may result from highly motivated signalers vibrating the nest substrate directly, as observed in studies of *A*. *mellifera* [19,23].

### Effects of Predator Type and Danger Level on Signal Characteristics

Graded individual alarm signals are well documented in vertebrates [3–5,35] but were not previously known in social insects. *Apis cerana* stop signals varied in two ways that reflected both predator threat and attack context (Fig 5). SS _large hornet_ consistently elicited a stronger inhibitory response than SS _small hornet_, whether in the context of nest or forager attacks. Large hornets elicited stop signals with a significantly higher fundamental frequency than small hornets in the context of forager attacks and nest attacks. Given how animal alarm systems commonly function [7,8], this frequency difference most likely reflects predator threat level, not predator identity. These higher frequency signals had a significantly stronger inhibitory effect on waggle dancing than the lower frequency signals (Fig 4), even though the waggle dancers in this bee-produced playback experiment did not encounter any predators at their feeder. In *A*. *mellifera*, bees are more sensitive (have a lower freezing response threshold) to higher-frequency stop signals [12]. Such increased sensitivity in *A*. *cerana* could explain the greater inhibitory effect of the higher frequency signals elicited by the larger hornet.

Stop signal pulse duration encoded attack context. Nest attacks resulted in significantly longer signals than forager attacks (Fig 5), and these longer signals were more effective at inhibiting nest departures (Fig 6B). In this experiment, signal receivers did not directly experience the predator because they were inside the nest. We cannot exclude the possibility that stop signal receivers smelled predator or honey bee alarm pheromone odors that may have entered the nest. However, the increased reluctance of receivers to leave the safety of the nest after receiving a SS _large hornet nest_ as compared to a SS _small hornet nest_ matches the increased signaling of senders, who penetrated deeper into the colony and produced more signals for the bigger threat, the large hornet (Fig 3B).

### Heat-balling Is Triggered by Predator Odor and Honey Bee Sting Alarm Pheromone

We originally hypothesized that SS _nest_ would activate defenders to heat-ball the hornet. This did not occur (Fig 6B). Instead, defenders were activated by honey bee sting alarm pheromone and the presence and smell of a live hornet (Fig 7). These data provide the first detailed experimental evidence for the role of bee alarm pheromone in heat-balling. The movements of the live hornet could have contributed to initial bee attraction, but once heat-balling is initiated, hornet motions are likely not detectable within the mass of buzzing bees (Fig 1B).

### New Stop Signal Functions and Graded Predator Alerts

Signals can evolve to serve new purposes and acquire new functions. We suggest that this has happened with stop signals in *A*. *cerana*. Both *A*. *cerana* and *A*. *mellifera* share stop signals that provoke the same proximate response—freezing of the receiver [12,19]—and have similar frequencies and durations. *A*. *mellifera* stop signals have an average fundamental frequency of 329 Hz and a mean duration of 170 ms [22], whereas *A*. *cerana* stop signals have somewhat higher mean fundamental frequencies of 502 and 550 Hz (small and large hornets, respectively) and durations of 178 and 258 ms resulting from forager and nest attacks, respectively (Fig 1). Towne [36] similarly reported that *A*. *cerana* stop signals have higher fundamental frequency (445 Hz, but within the range of what we measured) than those of *A*. *mellifera*. In both species, attacked foragers produce stop signals that inhibit waggle dancing [19,20]. However, *A*. *cerana* has formidable nest enemies—different species of giant Asian hornets—and has evolved stop signals that allow the colony to respond appropriately: inhibit recruitment for dangerous food (SS _forager_) or prevent departure from the safe nest when a predator is outside (SS _nest_). Moreover, SS _small hornet_ and SS _large hornet_ communicated graded levels of danger and elicited appropriate receiver responses. Such sophisticated alarm signals, previously only known in vertebrates, are therefore also used by insects.

## Materials and Methods

### General Methods

For all experiments, we used three *A*. *cerana cerana* colonies housed in observation hives in a room at Yunnan Agricultural University, Kunming, China from July to December 2012 (Experiment 1) and August to November 2014 and 2015 (all other experiments). Colonies 1, 2, and 3 each contained approximately 5,500 bees as determined by Liebefelder photographic estimation [37] during these time periods. The observation hives (55.4 x 17 x 64 cm) each contained two combs (43.5 x 23 cm) connected by a 25 cm long tube with a 2.2 cm inner diameter through the wall to the outside. Upon entering the nest, all bees were shunted by a wood and beeswax divider to one side of the lower comb, so that all forager activity could be viewed (method of [19]). We divided this lower comb into two equal-sized zones: closest to the nest entrance (Z1) and further from the nest entrance (Z2; Fig 3). Most waggle dances occurred in Z1 [38]. The glass covering one side of the nest was opened, allowing observers to record bee sounds. A door in the observation room allowed bees to escape, but, like, *A*. *mellifera* [18], the *A*. *cerana* foragers soon adjusted to the open hive and entered and exited through its entrance tube.

To record stop signals, an observer used a shielded microphone (Movo™ LV1 Lavalier Microphone) mounted on a thin metal rod connected to a RadioShack™ Mini Amplifier (model 277–1008) and recorded with manual audio gain on a Sony™ HDR-PJ790 camera. The nest monitor reported the number of waggle dance circuits and stop signals to a data recorder. Monitors were extensively trained to recognize the stop signal with a headphone splitter that allowed all experimenters to hear the microphone output. At the end of the first 15 trials, we reviewed the collected videos with each monitor to ensure identification accuracy. The stop signal is relatively easy to recognize because it has a characteristic sound frequency, is delivered by the signaler head-butting the receiver, and causes the receiver to immediately freeze for the duration of the signal. Once trained, monitors could detect the signal based upon its sound alone, as verified by video reviews and the Fourier analysis of identified stop signals.

Video data was viewed for additional measurements: the amount of time that foragers waited to unload their collected food (unloading wait time) and the total time each forager spent inside the nest per return trip (nest visit time). To record behaviors, the observer sequentially scanned three zones (Ent, Z1, and Z2) for 1 min each and then repeated the scans so that each zone was scanned four times during a trial. A scan consisted of placing the microphone near, but not blocking, the nest entrance (Ent) or holding the microphone approximately 1 cm away from bees on the comb and moving in a zigzag pattern, up and down, for zones Z1 and Z2. We conducted one trial per day with each colony.

Stop signal fundamental frequency and duration were measured with Raven Pro v.1.4 software from Fast Fourier Transform spectrogram analysis. For accuracy, we measured at least two stop signals per bee, but we calculated the average stop signal fundamental frequency and duration per bee to avoid pseudoreplication.

### Experiment 1: Predator Threat Levels

We trained 50 bees from each colony to a 30% sucrose solution feeder located 35 m from the focal colony by gently capturing departing foragers at the hive entrance in a vial and releasing them slowly at the training feeder (Experiment 1A). Bees from each colony were trained to a different location to avoid competition for the same food source. The feeder consisted of a 70 ml vial (8 cm high) inverted over a circular plastic disk with 18 feeding grooves through which the sucrose could flow. After being filled with sucrose solution, the vial was inverted on a blue plastic square. Each feeder could accommodate 30 foragers without crowding. We then removed the training feeder and set out three identical feeders spaced 30 cm apart and equidistant from the focal colony. We attached live hornets (see below) to wires positioned 10 cm above each feeder. The control feeder had only a clean, bare wire. Foragers then chose between the three feeders, whose positions were randomly swapped every 5 min. We counted the total number of bees landing to feed during a 10 min trial (only one trial conducted per day, maximum of 11.4 bees/min). Foragers could recruit without constraint and, thus, forager numbers reflected colony allocation decisions. Upon reaching the array, foragers chose individually, and, thus, the number of forager visits reflected both recruitment and individual feeder choices.

To determine the danger posed by hornet predation at the colony entrance, we presented hornets (large or small) at the nest entrance (Experiment 1B). Bees rapidly heat-balled the hornet, and, after 5 min, we placed the entire ball inside a plastic bag and allowed the bees to escape one-by-one from a small opening. We counted the number of escaping bees and the number of dead bees that remained in the bag. We used each hornet only once. To estimate the minimum surface area that heat-balling bees would need to cover to completely encapsulate a hornet, we calculated the surface area of the smallest ellipsoid into which the hornet would fit.

### Experiment 2: Effect of Feeder Attacks on Individual and Colony-Level Behaviors

We used the different hornets to attack foragers trained to a feeder filled with 2.5 M sucrose solution (65% sucrose w/w) and placed about 50 m away from the nest. To facilitate training, we added 3 μl of citral (Jinjingle Inc, Shanghai, China) to a circular piece of filter paper placed under the feeder [14]. We recorded the responses of individually tracked bees inside the nest (individual responses: Experiment 2A; colony-level responses: Experiment 2B). To allow accurate data recording, individual and colony-level responses were measured in separate trials.

We marked all trained foragers with numbered tags (Opalich Zeichenplättchen) attached to their thoraces with resin to identify them and verify their colony origin. We trained approximately 20 foragers, resulting in approximately ten bees foraging at any given time without crowding on the feeder. We removed additional foragers with an aspirator to maintain a consistent level of visitation. Other bees were allowed to feed and return to the colony. There was no fighting between bees from different colonies (a trigger for stop signals [18]) because all bees were marked, and we immediately captured any bees that came from the wrong colony. To avoid depleting the colony of foragers, we released all captured bees at the end of all trials on each day. We trained a new set of bees for each trial.

To test the effect of predator attacks on stop signaling, we captured foraging *V*. *velutina* (small) and *V*. *mandarinia* (large) hornets near our field site with an insect net and tethered a single hornet to the end of a 1 m long wood rod with a stiff wire wrapped between the hornet’s thorax and abdomen. The control treatment consisted of an identical rod with a similar wire, but with no hornet, held 1 cm away from a foraging bee. We used a different hornet for each trial. Hornet attack consisted of a hornet leg or mandible making contact with a bee. This contact typically lasted for a fraction of a second because the bees immediately fled. After each trial, we washed the rod with laboratory detergent followed by 100% ethanol to remove potential odors and let it dry in the sun for the remainder of the day. After use, hornets were returned to a holding cage and not reused for several days. We used each hornet approximately twice over all trials.

To determine individual responses (Experiment 2A), we recorded the within-nest behavior of focal foragers on return trips before and after attack. To determine the colony-level response (Experiment 2B), a tethered hornet attacked each of five foragers visiting the feeder. Each bee was attacked for approximately 2 s over a 1 min interval. If a bee flew away after a hornet attack, we waited for it to return before attacking it again. This was repeated for a trial duration of 12 min. The control treatment consisted of the experimenter replicating the motions of a clean rod close to, but not touching, foragers.

### Experiment 3: Effect of Nest Attacks

A *V*. *velutina* hornet typically attacks an *A*. *cerana* colony by hovering close to the nest entrance [30]. To simulate this, the experimenter held the tethered hornet close to, but not touching, bees entering and exiting the nest.

In a preliminary experiment using only the small hornet, *V*. *velutina*, we used two different controls. In the “rod-only control,” we tested the effect of the rod apparatus: the experimenter attached one end of a clean rod with a wire but no hornet on a tripod 10 cm away from the colony entrance. In the “no-rod control,” we simply measured nest behaviors in the absence of any rod at the entrance. The presence of the live hornet clearly disturbed the colony, as expected. Thus, each set of trials began with the no-rod control, followed by the rod-only control, and ended with the hornet treatment. We conducted ten trials with each colony for a total of 30 trials.

The results of this preliminary experiment with two control treatments were essentially the same as the results of the full experiment with both large and small hornets (Fig 3). Small hornet attacks increased stop signaling (treatment: *F*_2,85_ = 28.19, *p* < 0.0001), and more stop signals were produced in the nest entrance (location: *F*_2,174_ = 28.99, *p* < 0.0001), leading to a significant interaction between treatment*location (interaction: *F*_4,174_ = 18.97, *p* < 0.0001). Colony accounted for 8.2% of model variance. Just as in our final experiment, small hornet attacks at the colony entrance had no effect on colony waggle dancing (NS). In addition, there were no significant differences in waggle dancing or stop signaling elicited by our no-rod or rod-only controls. In our full experiment with large and small hornets, we therefore used just the rod-only control, which we hereafter call the “control.”

In this full experiment, we began each trial with the rod-only control followed by attacks using the small hornet or large hornet. With each colony, we conducted one trial in the morning and one trial in the afternoon, randomly alternating the hornet species used in each trial. Cleaning and hornet usage were the same as in Experiment 2.

### Experiment 4: Effect of Stop Signals on Waggle Dancers for Natural Food Sources

We randomly selected waggle dancers recruiting for natural resources (pollen or nectar) and recorded the complete visits of these foragers inside the nest, beginning each recording when a bee entered the nest and ending it when it left. We noted the dance circuits at which they received stop signals, since the dance circuit is the natural unit of replication and will vary in duration for food sources at different distances from the nest [14].

### Experiment 5: Effect of Stop Signals Induced by Different Predators: A Bee-Produced Stop Signal Playback

In *A*. *mellifera* [18] and in *A*. *cerana* (based upon our results), stop signalers will target waggle dancers that visit a feeder that smells identical to the one that the stop signaler is visiting. We therefore trained a separate group of marked foragers to a different feeder (see training methods in Experiment 2) that was never attacked by hornets and tested the response of these waggle dancers to stop signals elicited by large or small hornets. Randomly selected marked foragers were attacked with a hornet, while other marked foragers (non-attacked) were trained to a feeder located 3 m away so they would not have direct experience of attacks. Both feeders had the same citral scent so that stop signalers would target the waggle dancers from the non-attacked feeder [18]. When foragers from the non-attacked feeder returned to waggle dance, they received stop signals from attacked bees.

### Which Bees Produce SS _nest_ (Experiment 6) and How Do Receivers Respond (Experiment 7)?

To determine which types of workers produce SS _nest_ (Experiment 6), we presented treatments (large hornet, small hornet, or control) at the nest entrance. Hornets were slowly waved in front of the nest entrance but did not contact any bees. Guard bees and forager bees were dusted on the thorax with colored cornstarch dust (multiple colors, Henan Ruoya Company, China). We used colored cornstarch because it could be quickly applied, did not disturb the bees, and was clearly visible over the observation period. Guard bees were defined as bees that stayed at the colony entrance. Foragers were bees that brought back pollen or nectar into the nest. We recorded how many stop signals each bee produced over 5 min and then removed the bee to avoid scoring the same one twice.

To determine the effects of SS _nest_ upon signal receivers (Experiment 7), we marked equal numbers of guard bees and returning foragers with colored cornstarch as in Experiment 6, but without a treatment (large hornet, small hornet, or control) at the nest entrance. Marked bees, therefore, had no experience of a hornet outside the nest. Once bees were marked, we separately presented the treatments at the nest entrance. We then scanned each marked bee with the microphone to determine if it received a stop signal. Once a bee received a stop signal, we tracked it for 5 min to see if it stayed inside or exited the nest (as verified by a monitor standing outside the nest entrance), and then removed it to avoid potential pseudoreplication. We chose 5 min because our preliminary analysis showed that about 85% of marked bees would move toward the nest exit, in the absence of hornet attack, within 5 min. At the end of each trial, we removed the marked bees to avoid scoring the same bee in a subsequent trial.

### Experiment 8: What Causes Bees to Heat-Ball a Hornet?

We tested the effects of odor and predator presence upon heat-balling of *V*. *velutina*. There were three target types (clean 5 cm diameter filter paper, de-scented hornet, and live hornet). To half of these targets, we initially added a one-bee equivalent of *A*. *cerana* sting alarm pheromone from the focal colony, resulting in six different treatments. Each trial consisted of the successive presentation of each these six treatments, in random order. De-scented hornets were dead hornets that we repeatedly rinsed with dichloromethane to remove their cuticular odors and then thoroughly dried before presentation. This de-scenting procedure was effective because bees treated de-scented hornets and clean filter paper identically (Fig 7). We used a different hornet per trial.

The monitor then counted the number of bees that inspected the treatment (approached but did not land on the hornet or heat-ball). If a ball formed, this was scored as “yes.” We used only *V*. *velutina* because *V*. *mandarinia* nest attacks resulted in significantly higher mortality (Fig 1B), and we did not wish to excessively weaken the colonies.

### Statistics

For Experiments 1 and 8, we used a Univariate Repeated-Measures Analysis of Variance (ANOVA) with colony as the repeated measure, because we were determining colony responses to hornets at the feeder or at the nest entrance.

To analyze individual responses in Experiment 2, we compared the behaviors of each forager before and after it was attacked and, therefore, used Repeated-Measures Analysis of Variance (ANOVA, REML algorithm) with bee identity (the repeated measure), treatment (large or small hornet), and phase (not attacked or attacked). We initially included the trial time of day but removed it because there was no significant effect.

To analyze the effect of hornet attacks upon colony waggle dancing and stop signaling (Experiments 2 and 3), we used Repeated-Measures Analysis of Variance (ANOVA, REML algorithm) with trial (the repeated-measure) and treatment (control, small hornet, or large hornet).

For Experiments 4 and 5, we divided waggle dancing into deciles to facilitate comparing dances with different numbers of waggle circuits. We used chi-square tests to compare the distribution of stop signals received by waggle dancers with the null expectation of a uniform distribution (method of [20]). If stop signals cause bees to stop waggle dancing, stop signals should occur more often near the end of dances. We applied the Sequential Bonferonni correction (*k* = 2, SB indicates significance) because we tested each distribution separately and then tested if they differed from each other.

For Experiment 6, we used ANOVA and tested the effects of treatment (control, small hornet, or large hornet) and worker type (forager or guard). In Experiment 7, we used ANOVA and tested the effect of treatment (control, SS _small hornet nest_, and SS _large hornet nest_).

We also used ANOVA to determine the effect of predator type (large or small hornet) and attack context (feeder or nest attacks) upon the average stop signal fundamental frequency and duration per bee.

We log-transformed the number of waggle circuits, number of stop signals, unloading wait time, unloading time, and nest visit time to meet parametric assumptions. We used Tukey’s HSD test to compare between treatments. Colony was a random effect in all models unless otherwise specified (Experiments 1 and 8). All other effects were fixed. Nonsignificant results (*p* > 0.05) are reported as NS, unless the *p*-values are close to 0.05.

Data is deposited in the Dryad repository: http://dx.doi.org/10.5061/dryad.cf426 [39].
